# Fluoroscopically calibrated 3D-printed patient-specific instruments improve the accuracy of osteotomy during bone tumor resection adjacent to joints

**DOI:** 10.1186/s41205-024-00216-z

**Published:** 2024-04-24

**Authors:** Chen Wang, Siyi Huang, Yue Yu, Haijie Liang, Ruifeng Wang, Xiaodong Tang, Tao Ji

**Affiliations:** 1 Peking University People’s Hospital, Musculoskeletal Tumor Center, Beijing, China; 2 LDK Medical Co., Ltd., R&D, Beijing, China

**Keywords:** 3D printed patient-specific instrument, Joint-preserving surgery, Bone tumor, Accuracy

## Abstract

**Background:**

Inadequate surface matching, variation in the guide design, and soft tissue on the skeletal surface may make it difficult to accurately place the 3D-printed patient-specific instrument (PSI) exactly to the designated site, leading to decreased accuracy, or even errors. Consequently, we developed a novel 3D-printed PSI with fluoroscopy-guided positioning markers to enhance the accuracy of osteotomies in joint-preserving surgery. The current study was to compare whether the fluoroscopically calibrated PSI (FCPSI) can achieve better accuracy compared with freehand resection and conventional PSI (CPSI) resection.

**Methods:**

Simulated joint-preserving surgery was conducted using nine synthetic left knee bone models. Osteotomies adjacent to the knee joint were designed to evaluate the accuracy at the epiphysis side. The experiment was divided into three groups: free-hand, conventional PSI (CPSI), and fluoroscopically Calibrated PSI (FCPSI). Post-resection CT scans were quantitatively analyzed. Analysis of variance (ANOVA) was used.

**Result:**

FCPSI improved the resection accuracy significantly. The mean location accuracy is 2.66 mm for FCPSI compared to 6.36 mm (*P* < 0.001) for freehand resection and 4.58 mm (*P* = 0.012) for CPSI. The mean average distance is 1.27 mm compared to 2.99 mm (*p* < 0.001) and 2.11 mm (*p* = 0.049). The mean absolute angle is 2.16° compared to 8.50° (*p* < 0.001) and 5.54° (*p* = 0.021). The mean depth angle is 1.41° compared to 8.10° (*p* < 0.001) and 5.32° (*p* = 0.012). However, there were no significant differences in the front angle compared to the freehand resection group (*P* = 0.055) and CPSI (*P* = 0.599) group. The location accuracy observed with FCPSI was maintained at 4 mm, while CPSI and freehand resection exhibited a maximum deviation of 8 mm.

**Conclusion:**

The fluoroscopically calibrated 3D-printed patient-specific instruments improve the accuracy of osteotomy during bone tumor resection adjacent to joint joints compared to conventional PSI and freehand resection. In conclusion, this novel 3D-printed PSI offers significant accuracy improvement in joint preserving surgery with a minimal increase in time and design costs.

**Supplementary Information:**

The online version contains supplementary material available at 10.1186/s41205-024-00216-z.

## Background

With the development of diagnostic imaging, surgical techniques, and adjuvant therapies, the application of joint-preserving surgery in the treatment of primary malignant bone tumors is gradually increasing [1]. The epiphyseal area around the knee is a common site for primary malignant bone tumors [2], making wide resection adjacent to the joint necessary. Joint-preserving surgery allows patients to retain their original anatomic joint and function, which can be performed on selected patients with specific indications [1, 3]. The goal of surgery is to achieve R0 resection while preserving uninvolved metaphyseal bone and joint surfaces [4]. For pediatric patients whose bones are immature, joint sacrifice may cause various complications related to growth [5]. Therefore, surgical accuracy is crucial in joint-preserving surgery for bone tumors.

With the development of imaging techniques, surgeons can theoretically identify the precise borders of the primary bone tumor preoperatively [6] using CT and MRI data. Subsequently, the surgeon formulates a preoperative plan and performs a planned resection during surgery. However, mounting evidence underscores that several factors, encompassing surgical instruments and the surgical settings, can contribute to the inaccuracies during the resection [7], making it challenging to conduct preoperative planning accurately, even for highly experienced surgeons.

Currently, 3D-printed patient-specific instruments (PSI) have been introduced to increase the accuracy of osteotomy in the orthopedic field including bone tumor resection [8–11]. Combining preoperative imaging data and 3D printing technology, surgeons and engineers generate personalized custom instruments matching the patients’ bone surface, which can be placed on the bone surface intraoperatively, mainly by surface profiling. With the assistance of PSI, surgeons can precisely conduct preoperatively planned osteotomy at specific sites, and angles [8, 12]. However, the current PSI still has some limitations. Inadequate surface matching, variation in the guide design, and soft tissue on the skeletal surface may make it difficult to accurately place the PSI exactly to the designated site, leading to decreased accuracy, or even errors [8, 13, 14].

To address the shortcomings of the regular PSI, we designed a fluoroscopically calibrated 3D-printed patient-specific instrument (FCPSI) to improve the accuracy of PSI placement during surgical procedures. Basically, the concept was to introduce certain length markers, represented by metallic wires, which were determined by the relationship between the planned osteotomy site and joint surface or certain bony structures. The relationship can be confirmed by fluoroscopy during surgery. A typical FCPSI contains calibrated markers oriented in different directions and positions, thus serving as fluoroscopic and positional references. Intraoperatively, the surgeon first positions the FCPSI and captures an accurate anteroposterior (AP) view confirmed by the AP-calibrated marker(s) which usually presents as a dot on a true AP fluoroscopy. Subsequently, utilizing the position of the markers used to calibrate the osteotomy position in the image as a guide, the FCPSI’s placement is adjusted until the markers align consistently with the surgical plan. If a metaphyseal osteotomy is planned at a certain distance from the joint surface level, a marker with such length (distance) is embedded in the jig with one end being placed exactly parallel to the distal bony condyle. Ultimately, the FCPSI is placed and secured with K-wires to achieve a stable position, enabling accurate execution of the osteotomy plan. In order to ascertain the accuracy of the FCPSI, a simulated resection experiment was conducted to explore the accuracy of osteotomy adjacent to the knee joint using FCPSIs compared to both freehand resection and PSI without fluoroscopic calibration. We attempted to address the following questions: (1) In comparison to conventional PSI and freehand resection, what surgical accuracy can FCPSI achieve? (2) Does the utilization of FCPSI can achieve more stable osteotomy?

## Methods

### Experimental models and preoperative planning

**Fig. 1:**
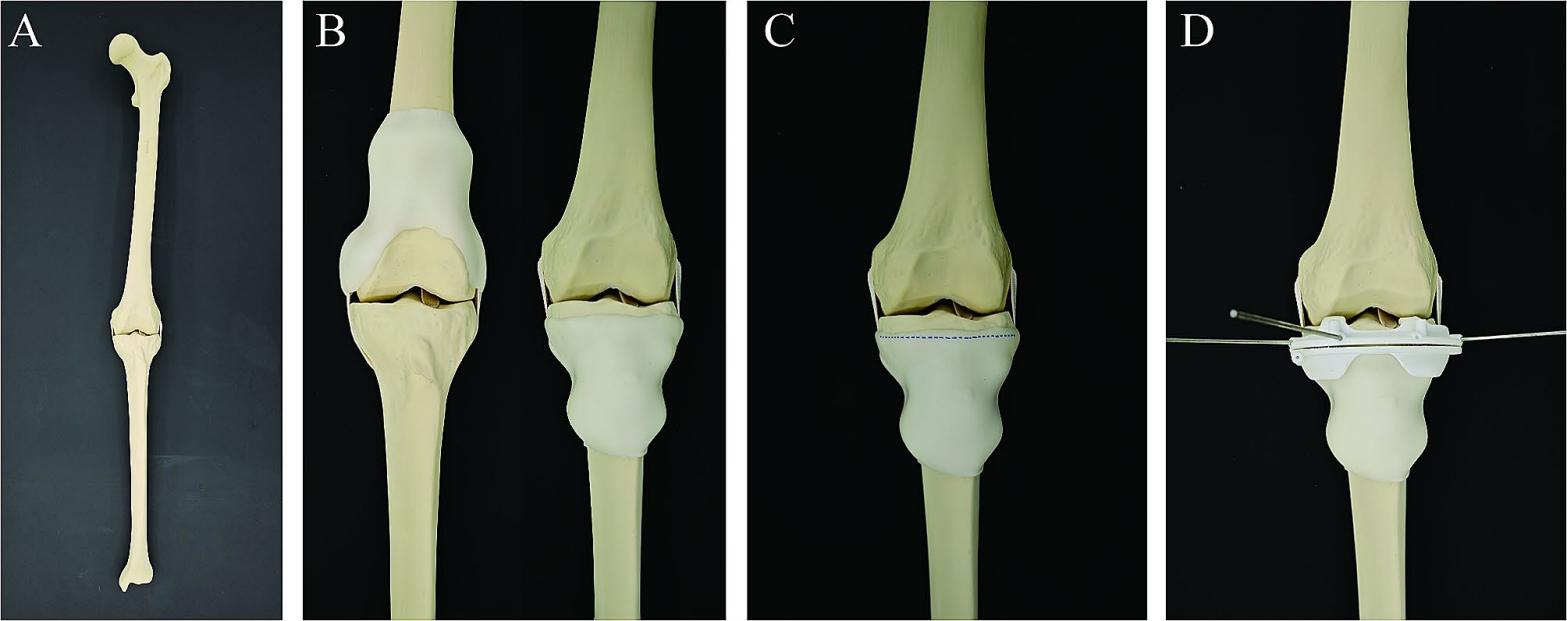
(**A**) The bone model used in the experiment is shown. (**B**) The bone models with the silicone model are shown. (**C**) In the manual resection group, the surgeon determined the position of the osteotomy line with the help of rigid and flexible rulers and labeled the model. (**D**) In the conventional PSI group and novel PSI group, the surgeon used different types of PSIs, respectively, and fixed them in the optimal position with appropriate methods. After that, the surgeon conducted the osteotomy

The experiments were conducted using nine synthetic left knee bone models (SYNBONE AG, Switzerland) consisting of the femur, tibia, medial collateral ligament, lateral collateral ligament, and the anterior and posterior cruciate ligament (Fig. 1A). The bone models were CT-scanned (layer thickness 0.625 mm, pixel size 0.3 mm) for DICOM files. Then CT images were used for preoperative resection planning with biomedical engineering software (Mimics 21.0; Materialise, Leuven, Belgium), and then the models’ computer-aided design (CAD) files were exported. Tumors were simulated in the distal femur and proximal tibia, which are adjacent to the knee joint, a common site for primary bone tumors. Then, two CAD files of 50 mm spheres representing the virtual tumors were placed at the distal femur and proximal tibial epiphysis.

**Fig. 2:**
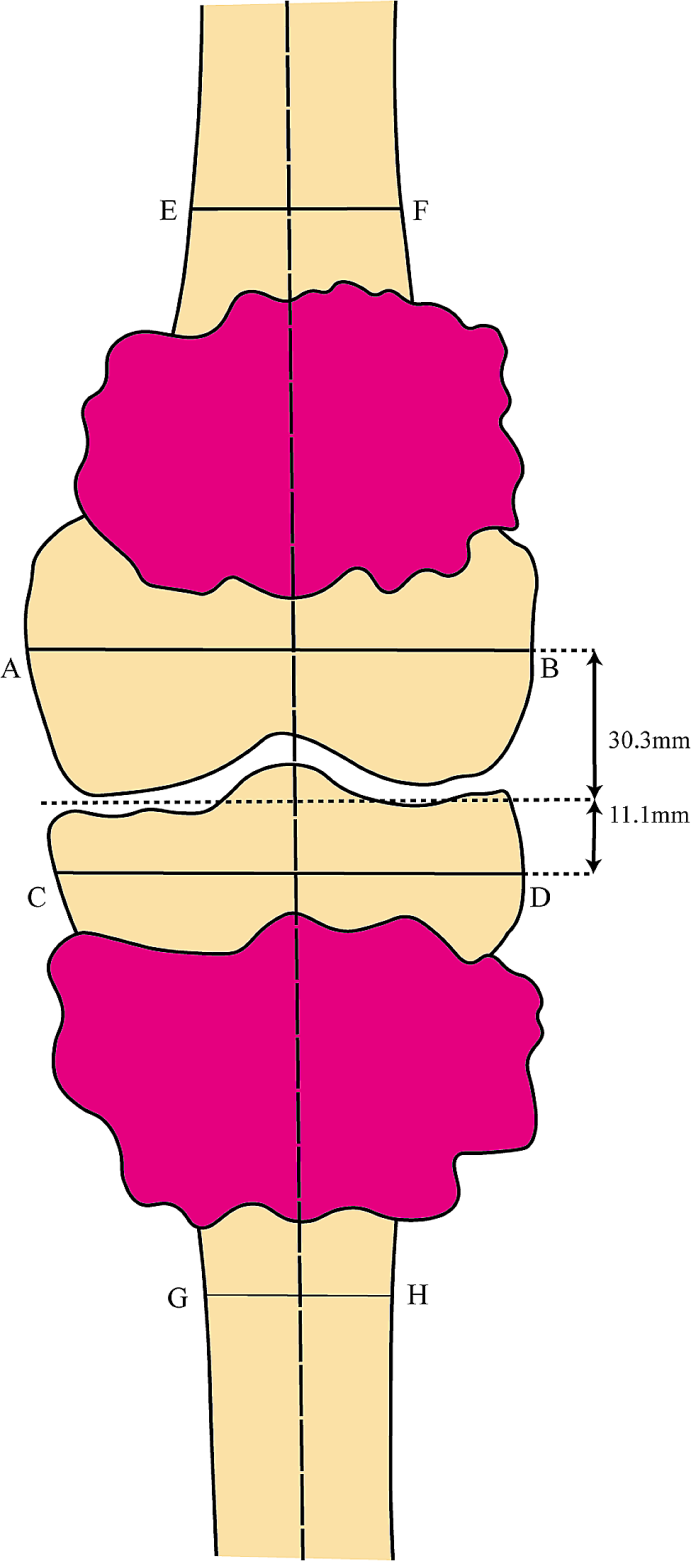
Illustrations based on the shapes of the experimental models. Simulated tumors are marked in red. The resection plans for the femoral side tumors are the osteotomy lines **AB** and **EF**, and for the tibial side tumors are the resection plans **CD** and **GH**. To compare the accuracy, we chose the resection planes **AB** and **CD**, which are more difficult for osteotomy. The center of the joint space on both sides was taken as the reference standard for the location of the resection plan

We conducted the tumor resection adjacent to the epiphysis simulating a joint-preserving surgery. Tumor resection protocols were formulated in the femur and tibia, each consisting of both proximal and distal resection plane planes (Fig. 2). The distances between the resection planes and the joint surface were 30.3 mm in the medial and lateral for the femur, and for the tibia were 11.0 mm, respectively. Both planned planes were parallel to the articular surface. The resection plane adjacent to the joint is critical to preserving the joint, as inadequate resection results in poor tumor margins, while excessive resection results in challenges in fixation during reconstruction and may cause complications after surgery. The present study was designed to evaluate the accuracy of osteotomy at the epiphysis side.

During actual procedures, there are soft-tissue masses enveloping the bone, and this may affect the placement of the surgical jig accurately for a non-calibrated PSI. To fully simulate the situation of bone tumor resection, a silicone model (Shore A hardness 10 degrees) was designed to encapsulate the affected femur or tibia to simulate the soft-tissue mass (Fig. 2B).

### Osteotomy

According to the methods of osteotomy, three groups were designed, which were free-hand, regular PSI without calibration, and FCPSI, respectively. A regular oscillating saw was used, and the details of the three groups are described below.

**Fig. 3:**
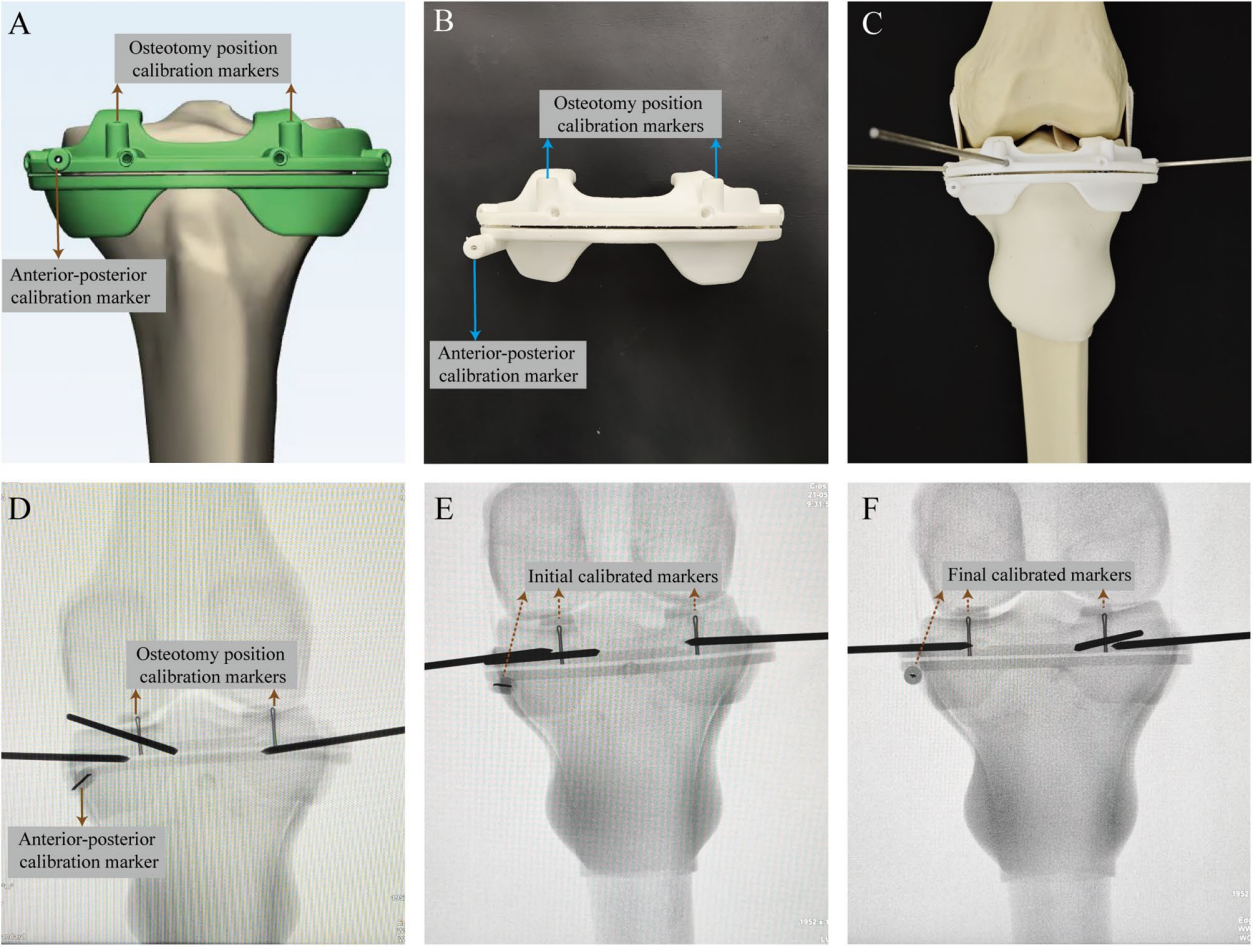
Illustration of FCPSI Design and application process. (**A**) (**B**) The metallic wires in different directions are added into the regular design as fluoroscopy calibration markers. According to the preoperative plan, an AP marker should be presented as a point on the AP view to achieve a true AP view on fluoroscopy. Then OP marker served as a reference to accurately place the guide on the coronal plane. The distal end of an OP marker should be aligned with the joint surface (the line between the distal end of medial and lateral condyles). (**C**) The FCPSI was initially assembled with the bone model. (**D-F**) Subsequently, the surgeon acquired a true AP X-ray image of the FCPSI. The position of C-arm was adjusted to achieve a position where the AP marker appears as a dot. With meticulous calibration to the optimal position (**F**), all markers adhered precisely to the preoperative planning as shown in (**A**)

(1) Freehand resection: In the freehand resection group, surgeons first reviewed CT images and the preoperative plan. They then obtained a printout of the resection plan’s coronal view, which included simulated tumor positions, osteotomy planes, and distances of anatomical landmarks. During surgery, surgeons creatively translated the 2D images into a 3D surgical situation and determined the osteotomy plane’s position with respect to anatomical landmarks. After precise measurement using standard equipment, the surgeon marked the osteotomy line on the model and confirmed its position with a ruler to verify the distance from the osteotomy site to the joint surface (Fig. 1C). Finally, an oscillating saw was used to complete the bone cut.

(2) Conventional PSI resection: Routinely, a PSI was designed and prepared according to the preoperative plan [8]. The design was conducted using engineering software (Mimics 21.0; Materialise, Leuven, Belgium). The bone contact surface of the PSI was matched to the surface profile of the bone with a slot to guide the saw blade during cutting. After completing the guide design, the PSI, made of nylon material, was fabricated using a selective laser sintering (SLS) 3D printer. Before osteotomy, the surgeon placed the PSI appropriately and then fixed it to the bone surface with K-wires (Fig. 1D). The placement of the PSI is determined by the surgeon’s experience, without additional tools. The oscillating saw was used to conduct the osteotomy.

(3) Fluoroscopically Calibrated PSI resection: We further designed the fluoroscopically calibrated PSI (FCPSI) using a similar design approach to the standard PSI (Fig. 3A, B). To calibrate the position of the PSI, we have incorporated sagittal and vertical metallic wires (markers) into the conventional PSI setup. For clarity, we designate the wire running along the anterior-posterior direction as the Anterior-Posterior calibration marker (AP marker). The wire aligning with the cephalocaudal direction is named as the Osteotomy Position calibration marker (OP marker) (Fig. 3A, Additional file 2). In our design, the AP marker aligns with the anterior-posterior direction on the sagittal view and should appear as a point in the AP view X-ray image. The OP marker placed cephalocaudal direction on coronal view, with its end intended to align with the corresponding joint surface (line of distal condyles) on the AP X-ray image. During the surgical procedure, the surgeon can assess the placement of the PSI by observing the imaging condition of the AP marker and the distance between the end of the OP marker and the joint surface.’

Before the resection procedure, the surgeon received instructions on how to use FCPSI. During surgery, the surgeon first placed the FCPSI at the appropriate position same as that of the regular PSI group (Fig. 3C). A C-arm was used to take an anterior-posterior (AP) view of the bone model (Fig. 3D). The position of the C-arm was adjusted to find a true AP view with the AP-calibrated marker appearing to be a dot. Subsequently, tiny adjustments of the jig were made on the coronal plane to place the distal end of the OP markers at the exact level of the distal bony condyle line (joint surface), ensuring an accurate determination of the osteotomy slot in accordance with the preoperative plan (Fig. 3E, F).

### Accuracy evaluation

To assess the accuracy of the three groups, all the resected specimens (layer thickness 0.625 mm, pixel size 0.3 mm) underwent CT scans. The 3D images of each model were created by engineering software (Mimics 21.0; Materialise, Leuven, Belgium). Then we merged the postoperative 3D models with the preoperative plan models. Then, the 3D model was converted into a closely distributed set of discrete coordinate points with reverse engineering software (Geomagic Studio 2014; 3D Systems Corporation, Rock Hill, SC, USA). Due to the gaps in cancellous bone, the points where cancellous bone intersect with the cut surface were difficult to identify [15]. Therefore, the set of discrete coordinate points at the cortical edges of the osteotomy was used to calculate the best-fit plane using the least squares method. Subsequently, the geometric relationship between the set of cortical edge data points (and the best-fit planes) and the target planes was evaluated for each bone model. In this study, the following definitions were used to describe the accuracy of the osteotomy.

**Fig. 4:**
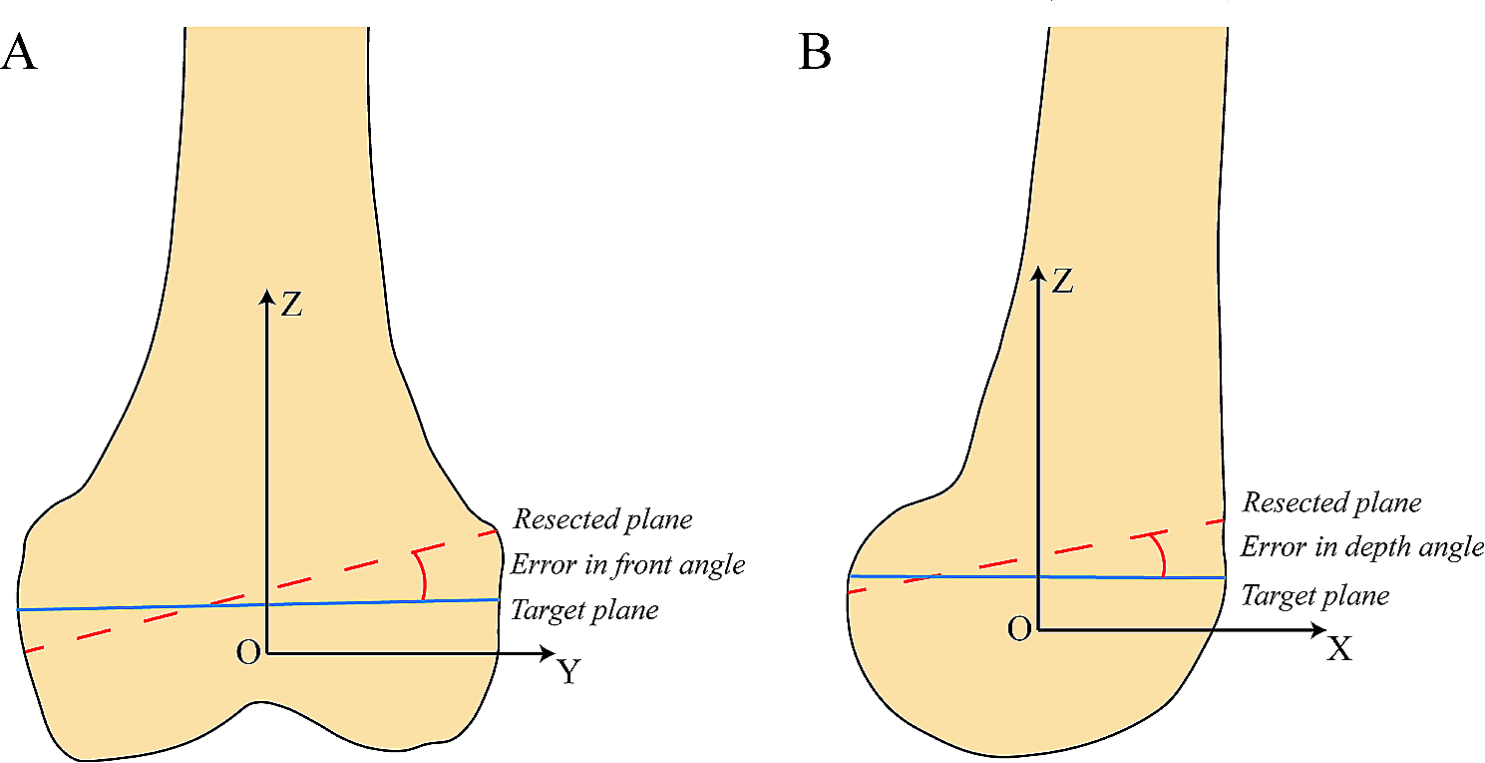
Description of accuracy evaluation metrics. Figure **A** shows the coronal plane section of the bone model. The red line is the intersection line between the actual resected plane and the coronal plane, and the blue line is the intersection line between the target plane and the coronal plane. According to our definition, error in front angle is the angle between two straight lines. Figure **B** shows the sagittal section of the model, and the error in depth angle is defined in the same way

(1) Location accuracy: The location accuracy refers to the maximum distance between the preoperatively planned and the actual osteotomy plane, as defined by the International Organization of Standardization (ISO). In this study, we computed the vertical distance between each point and the corresponding preoperative target plane based on the data set of coordinate points of the obtained cutting planes. The maximum of these distances represents the location accuracy.

(2) Average distance: The average distance is defined as the mean deviation between the preoperatively planned and actual osteotomy planes. To calculate the average distance, we take the mean of the absolute distances between each point in the aforementioned coordinate point set and its corresponding target plane.

(3) Error in front angle: The front angle is the rotation of the plane concerning the coronal axis (or yaw direction). The error in the front angle of the best-fit and target planes reflects the deviation between the two planes. The front angle is calculated by intersecting the best-fit and target planes with the anatomical coronal plane that contains the coordinate origin (Fig. 4A). The angle between the resulting projection target line and the corresponding resulting projection resection line is regarded as the error in the front angle.

(4) Error in depth angle: Similarly, the depth angle is defined as the rotation of the plane with respect to the sagittal axis (or pitch direction). The depth angle is calculated by intersecting the best-fit and target planes with the anatomical sagittal plane that contains the coordinate origin (Fig. 4B). The angle between the resulting projection target line and the corresponding resulting projection resection line is regarded as the error in depth angle.

(5) Absolute angle: The absolute angle is defined as the angle between the actual and target planes. The best-fit plane is adopted to calculate the angle.

(6) Percentage of successful resections within acceptable error: We determined the proportion of successful resections over all samples for different surgical errors. The maximum errors allowed for the successful excision of all samples in each group were also determined.

### Statistical analysis

The normality of the data was analyzed using the Shapiro-Wilk test, with all samples meeting the normality assumption. The homogeneity of variances was confirmed through the variance chi-square test for the groups. Consequently, the variation among the three groups in terms of location accuracy, mean distance, error in depth angle and anterior angle, and surgical margin were assessed using analysis of variance (ANOVA). In cases where significant differences were observed, post hoc analyses (LSD) were conducted to further investigate the specific contrasts. SPSS (Version 25.0; IBM Corp, Armonk, NY, USA) and MATLAB (Version R2022a; MathWorks Inc., Natick, MA, USA) were used for statistical data analysis. *P*-values less than 0.05 were considered statistically significant.

## Result

### Surgical accuracy

Eighteen cut planes are available to assess the resection accuracy of freehand resection, conventional PSI, and fluoroscopically calibrated PSI. Compared to the freehand resection group, the mean location accuracy was decreased from 6.36 mm to 4.58 mm in the conventional PSI group (*P* = 0.019) and 2.66 mm in the FCPSI group (*P* < 0.001). Correspondingly, the mean average distance was decreased from 2.99 mm to 2.11 mm in the conventional PSI group (*p* = 0.042) and 1.27 mm in the FCPSI group (*p* < 0.001). The mean absolute angle was decreased from 8.50° to 5.54° in the conventional PSI group (*p* = 0.039) and 2.16° in the FCPSI group (*p* < 0.001). The mean depth angle was decreased from 8.10° to 5.32° in the conventional PSI group (*p* = 0.062) and 1.41° in the novel PSI group (*p* < 0.001). Both the conventional PSI group (*p* = 0.019) and FCPSI group (*P* = 0.055) exhibited decreases in the front angle (Table 1).

**Table 1:** The mean deviation of resection planes from preoperative target planes

	Location accuracy (mm)	Average distance (mm)	Absolute angle (degree)	Front angle (degree)	Depth angle (degree)
Freehand	6.36	2.99	8.50	2.40	8.10
CPSI	4.58	2.11	5.54	1.02	5.32
FCPSI	2.66	1.27	2.16	1.30	1.41

**Fig. 5:**
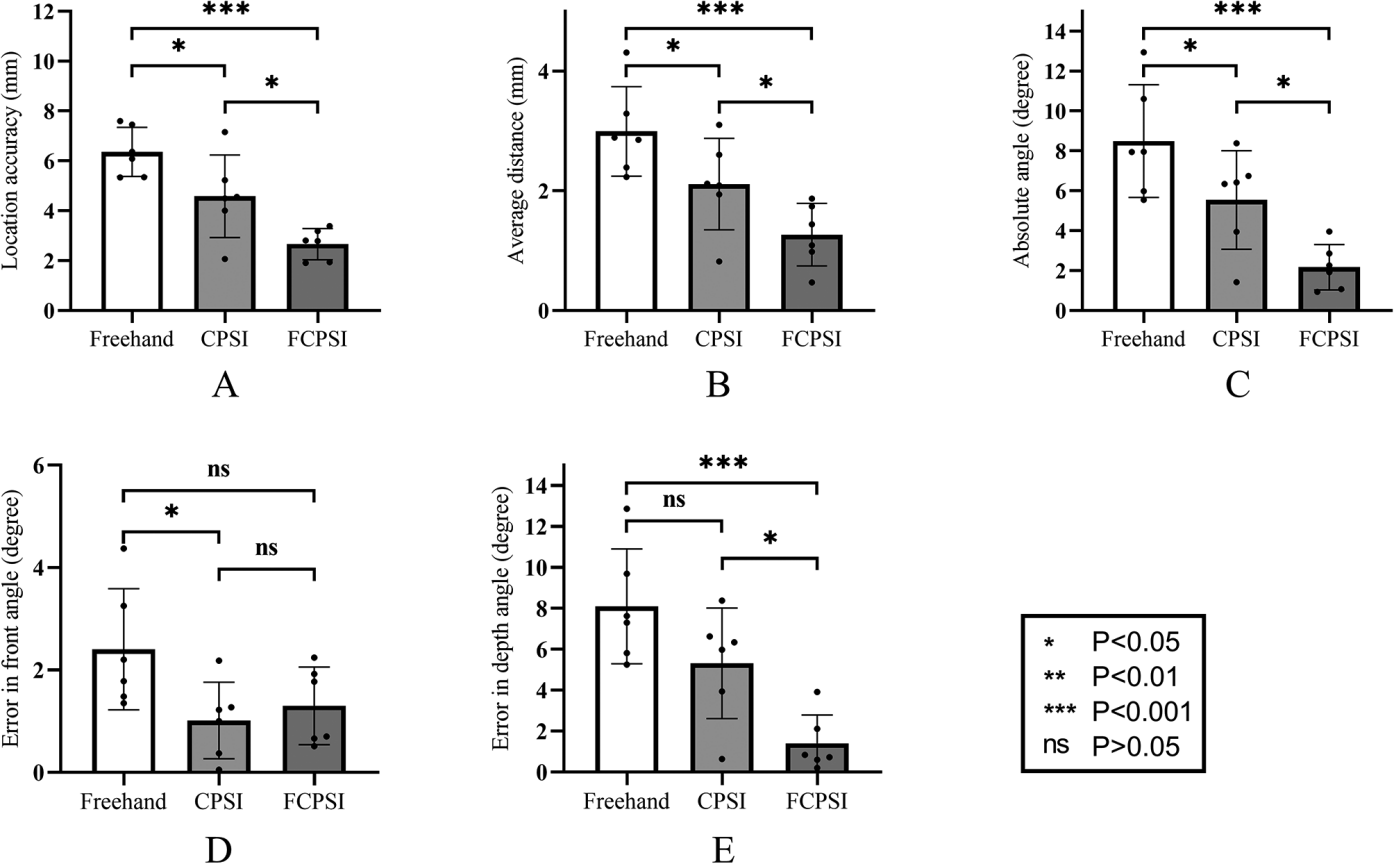
Comparison of localization accuracy (mm), average distance (mm), absolute angle (degree), front angle (degree), and depth angle (degree). The mean values for all study groups are shown, including the lower and upper limits of the 95% confidence interval

Compared with the conventional PSI group, the FCPSI group demonstrated improved accuracy of the osteotomy. The FCPSI exhibited significant enhancements in location accuracy (*P* = 0.012), average distance (*P* = 0.049), absolute angle (*P* = 0.021), and depth angle (*P* = 0.012). Conversely, no significant improvement or difference was observed between the two groups for the front angle (*P* = 0.599). Nonetheless, the error remained minor in both groups, measuring less than 1.30° (Fig. 5).

### Stability of accurate resections

The percentage of successful resections within a given surgical error margin was also determined (Table 2). The most significant maximum deviation from the preoperative plan was observed as 7.59 mm in the manual group, 7.15 mm in the conventional PSI group, and 3.38 mm in the fluoroscopically positioned PSI group, across all specimens and cut planes.

**Table 2:** Percentage of successful resections within accepted surgical error margin

Surgical error margin	2 mm	3 mm	4 mm	5 mm	6 mm	7 mm	8 mm	9 mm	10 mm
FCPSI	33.3%	66.7%	100%	100%	100%	100%	100%	100%	100%
CPSI	0.0%	16.7%	33.3%	66.7%	83.3%	83.3%	100%	100%	100%
Freehand	0.0%	0.0%	0.0%	0.0%	33.3%	66.7%	100%	100%	100%

## Discussion

Accurate reproduction of preoperative planning is crucial in bone tumor resection, especially the osteotomy adjacent to the joint. Insufficient tissue removal leads to positive margins, contributing to higher recurrence rates and poor prognosis [16]. In contrast, excision of excessive bone can damage anatomical structures including joints, nerves, and blood vessels, resulting in challenges in structural and functional reconstruction and even failure to preserve the joint. However, the accuracy of freehand resection and conventional 3D-printed PSI remains a subject of enhancement. In response, our study introduces the fluoroscopically calibrated 3D-printed PSI (FCPSI), which enables more precise placement with the assistance of the fluoroscopically positioned markers. By simulating the resection of distal femoral and proximal tibial bone tumors, our findings demonstrate that the FCPSI improves the precision of osteotomy in joint-adjacent bone tumor resections compared to freehand resection and conventional PSI.

Freehand resection remains the predominant approach for most bone tumor excision [17]. However, it has been demonstrated that freehand resection may engender significant surgical inaccuracies, even for experienced surgeons [7, 17]. 3D-printed patient-specific instruments, computer-assisted navigation systems, and robot-assisted surgery have been adopted in orthopedic surgery gradually [8, 15, 18]. These technologies enhance the accuracy of bone tumor surgery dramatically and the quality of surgical margins in limb-preserving surgery nowadays [12, 19–22]. Notably, the paradigm shift towards precision in medical treatment and advancements in medical technology have fostered the adoption of joint-preserving surgery by surgeons [1, 3]. However, existing techniques exhibit limitations in cases of joint-preserving surgery, particularly in osteotomies adjacent to the joint. In these cases, the reckless utilization of insufficiently reliable tools may lead to catastrophic consequences. In response, we designed the fluoroscopically calibrated PSI to further enhance surgical precision, addressing the demands of these intricate clinical situations.

The experimental results show that fluoroscopically calibrated PSI has a significant improvement in location accuracy, average distance, absolute angle, and depth angle compared with manual resection and conventional PSI. In particular, the conventional PSI did not show statistically significant differences in average deviation, absolute angle, and depth angle compared with manual resection, which further demonstrates the advantages and necessity of fluoroscopically calibrated PSI in osteotomies adjacent to the joint.

The mean location accuracy of 6.36 mm observed in the freehand resection group exhibits a reduction compared to previous research [7, 17, 23]. However, it is noteworthy that the distances from the target plane to the articular surface were 31 mm in the femur and 11 mm in the tibia approximately. Thus, the error of 6.36 mm assumes considerable significance with respect to the preoperative plan of 11.1 and 30.3 mm, introducing an uncontrollable surgical risk. In parallel, the mean location accuracy observed in the conventional PSI group is 4.58 mm, slightly larger than previously reported in the literature [8, 12, 19, 22, 23]. The error is consistent with expectations considering the factors that make the operation more challenging. In the experiments, silicone models were designed to simulate intraoperative soft tissues. Additionally, the location of the osteotomy planes is adjacent to the articular surface rather than the long bone’s midportion, as is commonly seen in previous studies [8, 23–25].

The improvement in depth angle reflects the ability of the fluoroscopically calibrated PSI to reduce the rotation of the resection planes and the deviation of the plane’s entry and exit cuts, which indicates that the AP marker serves as an essential calibration marker. Through meticulous adjustment of the AP marker, until it appears as a point on the X-ray images obtained in the AP perspective, surgeons ensure the precise placement of FCPSI on the bone models with minimal coronal axis deflection. The enhancement in depth angle addresses the issues of inaccurate entry and exit cuts as reported in previous literature [23].

However, the results did not reveal any significant difference between FCPSI and CPSI in the front angle, indicative of rotation along the sagittal axis. The cumulative outcomes contain both systematic and random errors. Considering the slight magnitude of errors within FCPSI and CPSI groups (< 1.30 degrees), it is plausible that random errors may exert a predominant influence across the board. These minor errors may be related to the fact that the initial target angle was set at 0 degrees. The future design could introduce the complexity in accurate reproduction by increasing the front angle of the target plane, which would amplify the systematic error within each group and yield more accurate outcomes.

In addition to accuracy considerations, conventional technologies introduce a series of problems. Computer-assisted navigation systems require expensive hardware equipment and complex software, accompanied by lengthy and often inaccurate registration processes based on paired points or intraoperative CT images [18, 26, 27]. Moreover, previous studies have shown that computer navigation can take a long learning curve for surgeons [28]. In certain scenarios, the lack of proficiency can even have a negative impact on surgical accuracy [29]. Besides, an essential drawback of conventional PSI lies in the reduced accuracy in the placement of the PSI during the actual operation, attributed to the presence of soft tissues on the skeletal surface and the constraints on the surgeon’s visual field [8]. Therefore, more soft tissue has to be removed in order to place the PSI accurately.

Compared to existing technologies, fluoroscopically calibrated PSI presents considerable advantages. Building upon the inherent user-friendliness and short learning curve that traditional PSI already provides [30], fluoroscopically calibrated PSI improves the accuracy of PSI’s placement significantly through the ingeniously straightforward design of the fluoroscopy-guided positioning markers. The surgeon’s task is to take intraoperative X-ray images for evaluation and adjustment utilizing the X-ray machine, which is equipped in most operating rooms. By adjusting the PSI’s position with the assistance of markers, the surgeon ensures the accurate placement of the PSI with minimal soft tissue removal, leading to reduced structural damage and enhanced overall prognosis. In addition, the fabrication complexity and time cost of the FCPSI mirrors those of the conventional PSI, rendering the FCPSI amenable to widespread adoption. In conclusion, the FCPSI enhances surgical accuracy with a cost-effective and efficient design process.

The fluoroscopically calibrated PSI also contains some limitations. Firstly, similar to the conventional PSI, the FCPSI requires a preoperative planning, design, and printing process, which is time-consuming and mandates engineering expertise [30]. Moreover, this design technique may not be available to all engineering teams. Secondly, PSI only applies to a single operation for a specific patient and cannot be reused, contributing to augmented usage costs. Thirdly, the FCPSI may marginally extend the surgical time due to the calibration process. However, the increased time is acceptable in most surgical scenarios, given the improved surgical accuracy and overall prognosis. Fourthly, the process of adjusting the PSI’s fixation position may result in more holes created by the K-wires. Finally, the utilization of the FCPSI requires intraoperative X-ray images, which introduce a marginal amount of radiation that may adversely affect the patient [8].

The following limitations exist in this study. Firstly, the simulated resection experiments were conducted using a synbone model with a silicone model to simulate the soft tissue. Nevertheless, the experiments still differed from the actual surgical conditions [12]. In particular, it is important to highlight that while the location of the bone tumor was carefully planned, the experiments did not account for the extension of malignant bone tumors into soft tissues. This oversight directly influences the precise determination of surgical margins and cutting guides. Therefore, additional cadaveric experiments and clinical case studies are imperative to ascertain the precision and reliability of FCPSI on both bone and soft tissue margins. Secondly, the location accuracy in the experimental results was in the range of 2–8 mm. In contrast, the bone models were CT scanned with a layer thickness of 0.625 mm, potentially leading to inaccuracies in the calculations related to the resection plane. Thirdly, we did not replicate the location and shape of the tumor on the bone model, which is a critical reference marker for surgeons in surgical resection. However, the tumor’s shape was factored into the silicone model, mitigating the influence on the surgeon’s judgment to some extent. The adoption of personalized bone models using imaging data from real bone tumor cases could eliminate the effect completely [31, 32]. Fourthly, whether the FCPSI could offer superior error reduction compared to other techniques, such as computer-assisted navigation and robot-assisted surgery [22, 23] remains unexplored. However, it is certain that the FCPSI exhibits more scalability in terms of cost and operational difficulty. Fifthly, the study focused on the resection of bone tumors adjacent to the joint in this study, neglecting other sites where bone tumors are commonly found, such as the pelvis [19, 20, 33]. Consequently, a comprehensive evaluation of this technique’s applicability and refinement in diverse surgical scenarios is necessary.

The role of fluoroscopic localization in improving the accuracy of PSI has been well validated in current in vitro experiments. However, future clinical applications must take into account the influence of patient conditions, design processes, surgeons’ experience and preferences. Patient conditions including patient obesity, limited joint mobility, and joint deformities can impact imaging quality. One potential solution is to develop patient-specific visualization and localization strategies, such as designing markers tailored to each patient’s unique conditions for easier localization. Surgeons’ determination during procedures could also potentially affect outcomes. In the future, implementing quantifiable measurement schemes or digital image recognition programs could enhance the accuracy of marker position judgment. Additionally, when designing PSI, it’s crucial to consider factors beyond just osteotomy success, such as incorporating MRI data to compensate cartilage of joint surface and ensuring adequate soft-tissue surgical margins.

In the future, our intentions encompass further clinical case research to determine this new technique’s stability and accuracy. We also intend to explore how the FCPSI measures up compared to other techniques in terms of accuracy and the potential benefits resulting from its combination with other techniques [34]. For example, the FCPSI could provide augmented localization stability within a shorter operative time in combination with CAS based on intraoperative images. While our current study focused on experimental research of bone tumor resection adjacent to joints, the feasibility of applying the FCPSI in other anatomical sites is also within our future plan. However, considering the anatomical complexity of the pelvis and other sites, the current design and application methods may require further improvement. Corresponding developed techniques and the requisite scope of accuracy need to be further explored.

## Conclusions

We developed a novel 3D-printed PSI equipped with fluoroscopy-guided positioning markers for precise intraoperative localization to address the inaccuracy of the conventional PSI. Through simulated resection of bone tumors adjacent to the joint, our findings demonstrated the improved accuracy of the fluoroscopically calibrated PSI compared to both the freehand resection and the conventional PSI. The novel 3D-printed PSI offers significant accuracy improvement in joint preserving surgery with a minimal increase in time and design costs. Further cadaveric or clinical trials are necessary to evaluate the safety and accuracy in authentic clinical settings, thereby paving the way for the expanded clinical application of the FCPSI.

### Electronic supplementary material

Below is the link to the electronic supplementary material.

**Supplementary Material 1**: Original Data of Osteotomy

**Supplementary Material 2**: Introduction of Fluoroscopically Calibrated 3D-Printed Patient-specific Instruments (FCPSI)

## Data Availability

No datasets were generated or analysed during the current study.

## Abbreviations

PSI: patient-specific instrument
CPSI: conventional patient-specific instrument
FCPSI: fluoroscopically calibrated patient-specific instrument
CAS: computer-aided surgery
AP: Anteroposterior
AP marker: Anterior-posterior calibration marker
OP marker: Osteotomy Position calibration marker
