# Supplementary material for: Fluoroscopically calibrated 3D-printed patient-specific instruments improve the accuracy of osteotomy during bone tumor resection adjacent to joints
Source: 3D Print Med. 2024 Apr 24;10:15. doi: 10.1186/s41205-024-00216-z (PMC11041006; doi:10.1186/s41205-024-00216-z)
Supplement: Supplementary file 2 — Supplementary Material 2: Introduction of Fluoroscopically Calibrated 3D-Printed Patient-specific Instruments (FCPSI) [file 41205_2024_216_MOESM2_ESM.pdf]

Introduction of Fluoroscopically Calibrated 3D-Printed Patient-specific Instruments (FCPSI)

Step 1. FCPSI Design and Printing

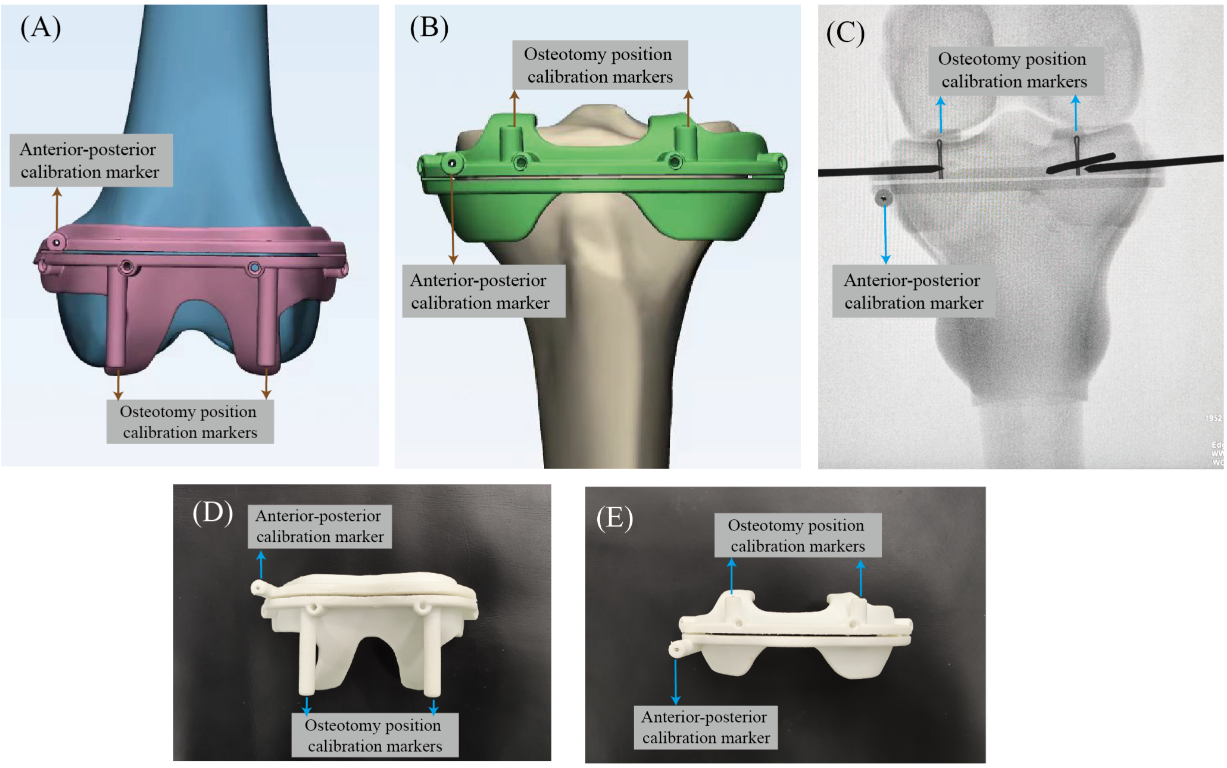

Figure S1. (A) (B) Design approach to FCPSI. (C) Position of markers in anteroposterior X-ray image. (D) (E) 3D-printed FCPSI models customized for the femur and tibia.

FCPSI introduces metallic wires at specific locations that assist in positioning compared to the design of conventional PSI. For clarity, we designate the wire running along the anterior-posterior direction as the Anterior-Posterior calibration marker (AP marker). The wire aligning with the cephalocaudal direction is termed the Osteotomy Position calibration marker (OP marker) (Figure S1 A, B). In our design, the AP marker aligns with the anterior-posterior direction on the sagittal view and should appear as a point in the anteroposterior (AP) X-ray image. The OP marker follows the cephalocaudal direction on coronal view, with its end intended to align with the corresponding joint surface in the AP X-ray image (Figure S2 C). During the surgical procedure, the surgeon can assess the placement of the PSI by observing the imaging condition of the AP marker and the distance between the end of the OP marker and the joint surface. This information enables further adjustments as necessary.

The design process is carried out using Mimics (21.0; Materialise, Leuven, Belgium). Subsequent to completing the guide design, the PSI, constructed from nylon material, is fabricated utilizing a selective laser sintering (SLS) 3D printer (Figure S1 D, E).

Step 2. Preoperative preparation Adjustments of C-arm

In the actual procedure, the knee model is first positioned on the operating table appropriately. To obtain an AP position X-ray image of the joint, adjustments are made to the perspective angle of the C-arm. These adjustments are refined until a clear AP X-ray image is obtained, typically characterized by distinct visibility of the joint space and bone edges.

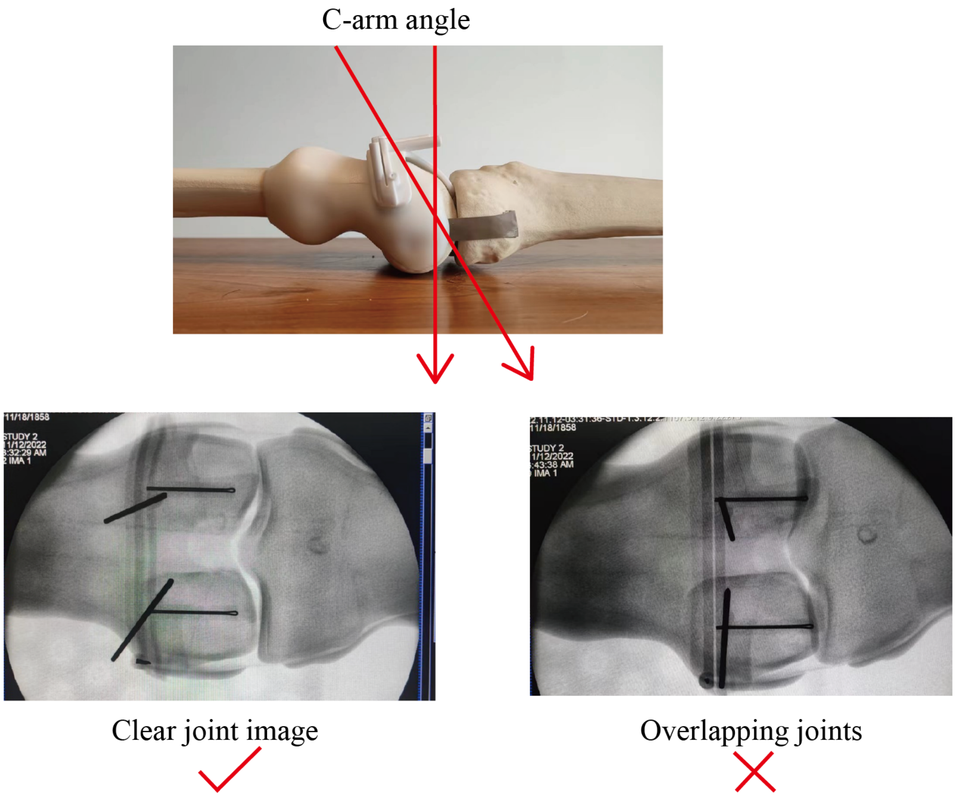

Figure 2. Different C-arm angles affect imaging quality and calibration accuracy

It's crucial to adjust the camera position of the C-arm X-ray machine to a proper angle during imaging. Failure to do so may compromise the imaging quality of the joint, leading to inaccurate calibration of the markers.

Step 3. Placement and adjustment of FCPSI

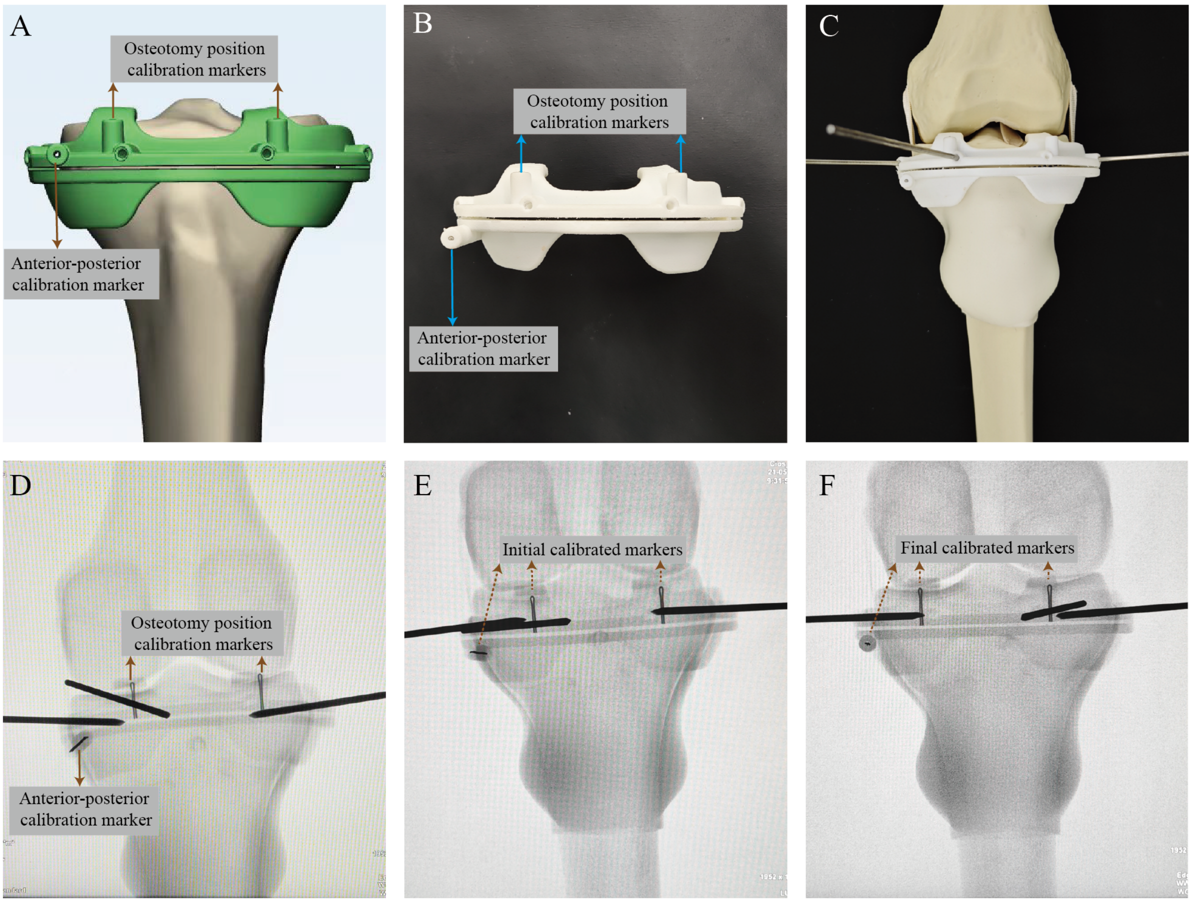

Figure 3. The adjustment process of FCPSI using the tibia as an example. (A) (B) The location of markers. (C) The initial placement of FCPSI. (D) The position of the markers during the initial placement, which notably deviates from the planned position, indicating placement inaccuracy. (E) The readjusted FCPSI, showing considerable improvement in position. (F) The final adjusted FCPSI, where the position of the developer filament now perfectly aligns with the plan.

The surgeon begins by positioning the FCPSI appropriately, akin to a standard PSI. The position of the FCPSI is then assessed through AP X-ray images obtained from the C-arm. As per our preoperative planning, AP marker should appear as a point on the AP X-ray images, while OP markers should align with the joint space. The surgeon accesses the placement of the FCPSI by the imaging condition of the AP marker and the distance between the end of the OP marker and the joint surface, and makes adjustments as necessary until it matches the intended position of the FCPSI.

Step 4 Osteotomy

Once the FCPSI has been properly positioned and secured with K-wires, the osteotomy can be performed using the appropriate surgical tools.
